# Long‐term risk of gastrointestinal cancers in persons with gastric or duodenal ulcers

**DOI:** 10.1002/cam4.680

**Published:** 2016-02-29

**Authors:** Kirstine K. Søgaard, Dóra K Farkas, Lars Pedersen, Jennifer L. Lund, Reimar W. Thomsen, Henrik T. Sørensen

**Affiliations:** ^1^ Department of Clinical EpidemiologyAarhus University HospitalAarhusDenmark; ^2^ Department of EpidemiologyGillings School of Global Public HealthUniversity of North CarolinaChapel HillNorth Carolina

**Keywords:** Epidemiology, Helicobacter pylori, neoplasm, peptic ulcer, risk

## Abstract

Peptic ulcer predicts gastric cancer. It is controversial if peptic ulcers predict other gastrointestinal cancers, potentially related to Helicobacter pylori or shared lifestyle factors. We hypothesized that gastric and duodenal ulcers may have different impact on the risk of gastrointestinal cancers. In a nationwide cohort study using Danish medical databases 1994–2013, we quantified the risk of gastric and other gastrointestinal cancers among patients with duodenal ulcers (dominantly H. pylori‐related) and gastric ulcers (dominantly lifestyle‐related) compared with the general population. We started follow‐up 1‐year after ulcer diagnosis to avoid detection bias and calculated absolute risks of cancer and standardized incidence ratios (SIRs). We identified 54,565 patients with gastric ulcers and 38,576 patients with duodenal ulcers. Patient characteristics were similar in the two cohorts. The 1–5‐year risk of any gastrointestinal cancer was slightly higher for gastric ulcers patients (2.1%) than for duodenal ulcers patients (2.0%), and SIRs were 1.38 (95% CI: 1.31–1.44) and 1.30 (95% CI: 1.23–1.37), respectively. The SIR of gastric cancer was higher among patients with gastric ulcer than duodenal ulcer (1.92 vs. 1.38), while the SIRs for other gastrointestinal cancers were similar (1.33 vs. 1.29). Compared with gastric ulcer patients, duodenal ulcer patients were at lower risk of smoking‐ and alcohol‐related gastrointestinal cancers. The risk of nongastric gastrointestinal cancers is increased both for patients with gastric ulcers and with duodenal ulcers, but absolute risks are low. H. pylori may be less important for the development of nongastric gastrointestinal cancer than hypothesized.

## Introduction

Peptic ulcer disease (PUD) is a common condition, leading to incident hospital contact in 2 per 1000 persons annually 1. Helicobacter pylori (H. pylori) colonizes both duodenal ulcers (approximately 80% 2) and to a lesser degree gastric ulcers (approximately 50% 3), and has been associated with increased risk of gastric cancer 4, 5. H. pylori also has been detected in nongastric gastrointestinal (GI) tissue, though this may reflect natural excretions rather than tissue colonization 6. Therefore, there is growing interest in a possible link between H. pylori infection, peptic ulcer, and risk of nongastric GI cancers 7, 8, 9. In addition, several lifestyle factors are associated with an increased risk of PUD and also affect cancer risk. Aspirin and other nonsteroidal anti‐inflammatory drugs (NSAIDs) increase PUD risk, particularly gastric ulcers 10, but may lower the risk of gastric and colorectal cancer 11, 12, 13. Tobacco smoking is an important risk factor for PUD 10 and is associated with the development of several GI cancers 14. Finally, alcohol overuse may increase risk of PUD, as indicated by the strong association between liver cirrhosis and pancreatic disease and increased PUD incidence 15. Alcohol overuse is similarly associated with several GI cancers 10, 14.

While H. pylori is highly prevalent among duodenal ulcer patients, use of NSAIDs, smoking, and alcohol‐related disease may be particularly frequent among gastric ulcer patients 15, 16, 17. Because of these possible differences, we hypothesized that gastric and duodenal ulcers may have different impact on the risk of GI cancers. We assessed absolute risks of gastric and other GI cancers among gastric and duodenal ulcer patients identified in Danish medical registries. We then compared their cancer risk with that in the general Danish population.

## Materials and Methods

### Data sources and study population

This registry‐based cohort study was based on the cumulative Danish population of approximately 7 million persons during the 1994–2013 period. The Danish National Health Service provides tax‐funded medical care to all Danish residents and guarantees free access to hospitals and outpatient clinics 18. The unique identifier assigned to every Danish resident allows linkage among Danish registries 19. We identified gastric and duodenal ulcer patients from the Danish National Patient Registry (DNPR), which has recorded all admissions to Danish hospitals since 1977 and all outpatient clinic visits since 1994. Diagnoses are classified according to the International Classification of Diseases (ICD), 8th revision (ICD‐8) until the end of 1993 and 10th revision (ICD‐10) thereafter 20. The main reason for diagnostic work‐up and treatment during a hospital contact is registered in the DNPR as the primary diagnosis, whereas other acute and chronic diseases or conditions are recorded as secondary diagnoses. We identified all patients with a first‐time hospital diagnosis (inpatient diagnosis or hospital outpatient clinic visit diagnosis) of gastric ulcer or duodenal ulcer during the period January 1994 to November 2013. We obtained information on reimbursed medications redeemed at Danish community and outpatient pharmacies from the Danish National Health Service Prescription Database (DNDRP), established in 2004 21. We obtained data on cancer diagnoses, classified according to ICD–10, from the Danish Cancer Registry (DCR), which has recorded incident cancers in Denmark since 1943 22.

All ICD‐codes and Anatomical Therapeutic Chemical (ATC) classification system codes used in this study are provided as online material (Appendix A1).

### Cancer

We linked all members of our study cohorts to the DCR to identify incident GI cancer cases (using ICD–10 codes). We excluded patients diagnosed with cancer (expect for non–melanoma skin cancer) before the date of PUD diagnosis. We then excluded cancers occurring during the first year of follow‐up to minimize inclusion of ulcers detected during diagnostic work‐up for cancer, as well as to exclude cancers detected in patients for whom an ulcer prompted further diagnostic work‐up. We examined the occurrence of the following GI cancers one or more years after PUD: oral and pharyngeal, esophageal, gastric, small intestinal, colon, rectal, anal, pancreatic, liver, and gallbladder and biliary tract. We considered oro‐pharyngeal, esophageal, gastric, colorectal, pancreatic, and liver cancers as tobacco‐ and alcohol‐related cancers 10, 14.

### Covariates

To characterize patients and address the potential for confounding and effect modification, we obtained information on coexisting diseases, proxy measures of lifestyle factors, and medication use before the hospital contact for ulcers. From the DNPR, we obtained information on chronic pulmonary disease (as a proxy for smoking), alcoholism‐related conditions (as a proxy for excess alcohol intake), severe liver disease, diabetes, obesity, and cardiovascular disease diagnosed at any time prior to or during the hospital contact for ulcer disease. We also obtained information on gastroscopies and lower endoscopies performed during the hospital contact for ulcer disease.

From the DNDRP, we obtained information on use (yes/no) of NSAIDs (including low‐ and high‐dose aspirin, selective cyclooxygenase‐2 inhibitors, and other NSAIDs); proton‐pump inhibitors (PPIs) and histamine H2‐receptor antagonists (H2‐blockers); and H. pylori‐eradication therapy (the combination of amoxicillin, clarithromycin, and metronidazole) in the year preceding the ulcer diagnosis. Information on drug use was available for patients diagnosed with PUD during 2005–2013, allowing 1 year time window of medication use for all patients.

### Statistical analysis

We followed each patient for occurrence of cancer starting 1 year after first hospital contact with a gastric or duodenal ulcer diagnosis until the date of death, emigration, or 30 November 2013, whichever came first. We computed distributions and frequencies of gender, age categories (≤39, 40–64, 65+), and the covariates. Median follow‐up (interquartile range, IQR) and median age (IQR) at inclusion were calculated.

We computed 1–5‐year absolute risks of cancer in patients with PUD, considering death as a competing risk 23, and censoring patients at the end of the study period. We used standardized incidence ratios (SIRs) as a measure of relative risks to compare the observed cancer incidence among patients with PUD with that expected in the entire Danish population 24. The expected number of cancer cases was estimated based on national cancer incidence rates by age (5‐year age groups), sex, and calendar year (5‐year periods). Confidence intervals (95% CIs) for SIRs were calculated assuming that the observed number of cancers followed a Poisson distribution, using Byar's approximation 25. We calculated SIRs for all GI cancers combined and individually and stratified by patient characteristics.

Because we were interested in detecting possible differences in characteristics and cancer risks between patients with gastric versus duodenal ulcers, all analyses were performed according to ulcer site. In a secondary analysis, we compared the observed cancer incidence among duodenal ulcer patients directly to that found among gastric ulcer patients (standardized on age, gender, and calendar year) 24.

All statistical analyses were conducted using the SAS statistical software package, v. 9.2 (SAS Institute, Cary, NC). The study was approved by Danish Data Protection Board (record number 1‐16–02–1–08 and 2012–41–0793). Danish registry data are generally available for research purposes and, according to Danish law, use of the data do not require informed consent.

## Results

We identified 119,212 patients with a first gastric or duodenal ulcer diagnosis between 1994 and 2013. Among all patients the 1‐year absolute risk of cancer was 2.3%. We then excluded 5672 patients diagnosed with cancer within the first year after ulcer diagnosis (oral and pharyngeal cancer: 84 cases; esophageal cancer: 106 cases; gastric cancer: 926 cases; small intestinal cancer: 100 cases; colon cancer: 676 cases, rectal cancer: 132 cases; anal cancer: 8 cases; pancreatic cancer: 404 cases; liver cancer: 144 cases; gallbladder or biliary tract cancer: 89 cases; other cancers: 3003 cases) and 20,399 patients with less than 1 year of follow‐up, yielding a total of 93,141 persons with a hospital‐based gastric or duodenal ulcer diagnosis. Among patients diagnosed with GI cancer in the first year of follow‐up, the prevalence of gastroscopy performed during the ulcer‐related hospital contact was 75% and the corresponding prevalence of lower endoscopy was 12%.

### Patient characteristics

Our final cohort of 93,141 peptic ulcer disease patients included 54,565 (59%) with a first gastric ulcer diagnosis and 38,576 (41%) with a first duodenal ulcer diagnosis. Patients with gastric ulcer were slightly older than patients with duodenal ulcer (median ages: 65 years vs. 63 years). After a 1–year induction period, patients with gastric ulcers and duodenal ulcers were followed for a median of 5 and 6 years, respectively. Most patients were diagnosed with an ulcer during an inpatient admission, the ulcer was the main reason for admission, and the diagnosis was gastroscopically confirmed (Table 1). Patients with gastric and duodenal ulcers were remarkably similar with respect to individual comorbid diseases, including a hospital‐based history of chronic obstructive pulmonary disease and alcoholism (Table 1). Recent use of NSAIDS and PPI or H2‐blockers was frequent among both groups of ulcer patients, and every 6–7th patient had H. pylori‐eradication therapy administered in the year preceding the diagnosis (Table 1).

**Table 1:** Characteristics of patients with gastric ulcer (*n* = 54,565) and duodenal ulcer (*n* = 38,576), Denmark, 1994–2013

	Gastric ulcer, *n* (%)	Duodenal ulcer, *n* (%)
Women	29,372 (54)	16,755 (43)
Median age, years (IQR)	65 (52–77)	63 (49–76)
Median follow‐up, years (IQR)	5 (2–10)	6 (3–12)
Calendar period		
1994–1998	16,730 (31)	13,954 (36)
1999–2003	14,723 (27)	11,384 (30)
2004–2008	13,550 (25)	8323 (21)
2009–2013	9562 (17)	4915 (13)
Type of ulcer diagnosis		
Primary	43,544 (80)	32,554 (84)
Secondary	11,021 (20)	6022 (16)
Type of admission		
Inpatient	36,426 (67)	27,571 (72)
Emergency room	4203 (8)	1283 (3)
Outpatient	13,936 (25)	9722 (25)
Comorbidities diagnosed prior to the ulcer		
Chronic obstructive pulmonary disease	4265 (8)	2878 (7)
Chronic alcoholism	4946 (9)	3605 (9)
Severe liver disease	911 (2)	559 (1)
Diabetes	4745 (9)	2936 (8)
Obesity	2660 (5)	1233 (3)
Cardiovascular disease	17,930 (33)	10,428 (27)
Endoscopies during the hospital contact for ulcers		
Gastroscopy	38,834 (71)	28,815 (75)
Lower endoscopy^a^	3698 (7)	2256 (6)
Medication use in the year preceding the ulcer^b^		
NSAIDs	12,378 (61)	6,793 (60)
PPI or H2‐blockers	8916 (44)	4480 (40)
Eradication therapy^c^	2558 (13)	1751 (16)

NSAIDS, Aspirin and other nonsteroidal anti‐inflammatory drugs.

a Specifically among patients diagnosed with colorectal cancer within the first year of follow‐up, the prevalence of lower endoscopy was 49% within 3 months and 77% within 1 year.

b Use of medications in the year preceding peptic ulcer diagnosis was only available for patients diagnosed between 2005 and 2013.

c Combination of amoxicillin, clarithromycin, and metronidazole.

### Gastrointestinal cancer in gastric and duodenal ulcer patients versus general population

**Figure 1:**
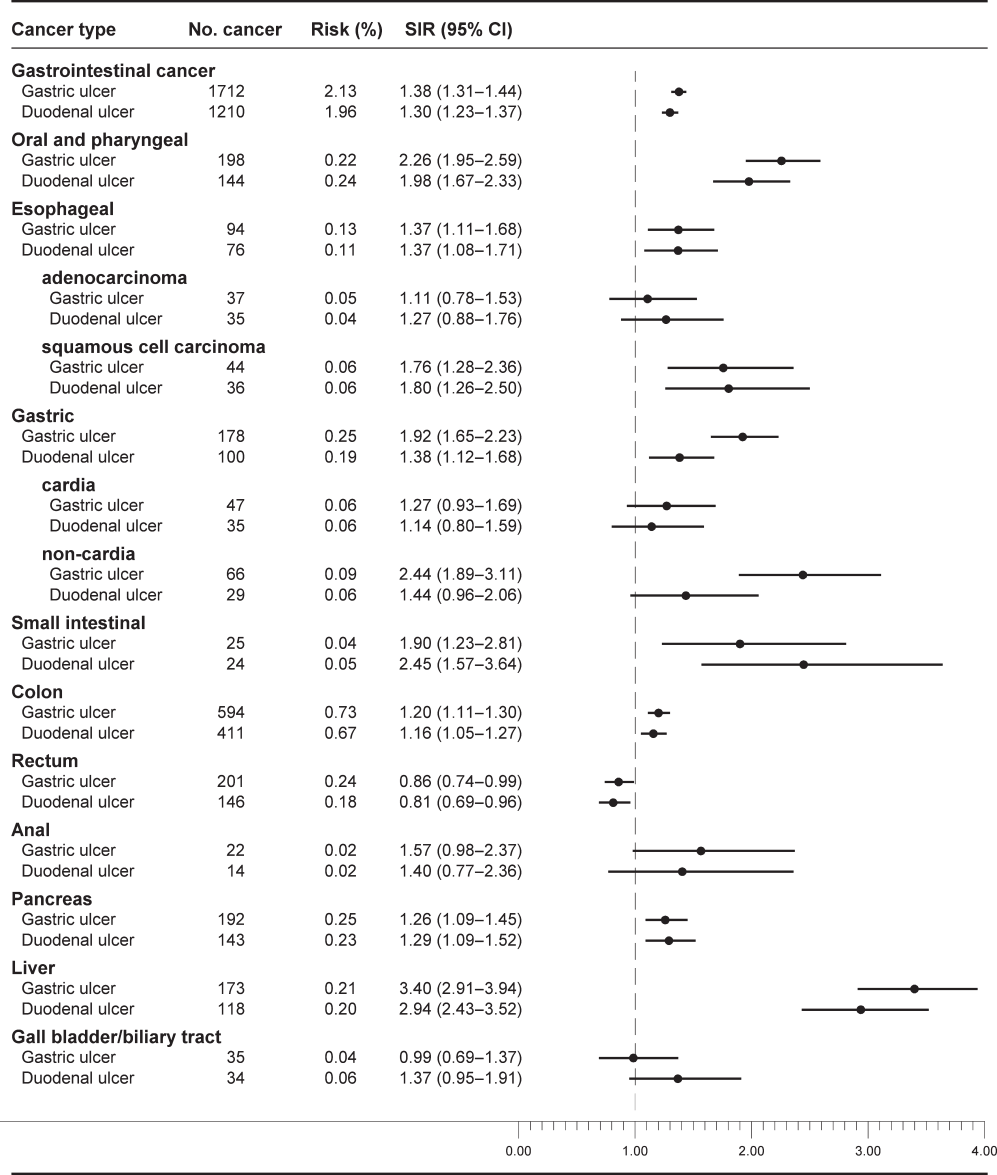
Number of gastrointestinal cancers; 1–5‐year absolute cancer risk (excluding cancer diagnosed during first year), treating death as a competing risk; and standardized incidence ratios (SIRs) of cancer one or more years after the first hospital contact for peptic ulcer, stratified by ulcer site.

During follow‐up, a total of 1712 GI cancers were diagnosed among patients with gastric ulcers, and 1210 GI cancers among patients with duodenal ulcers. The absolute 1–5‐year risk of any GI cancer was 2.1% for patients with a gastric ulcer and 2.0% for patients with a duodenal ulcer. Site‐specific cancer risks were similar in the two ulcer cohorts (Fig. 1). The SIR of any GI cancer compared with the general population was 1.38 (95% CI: 1.31–1.44) for patients with gastric ulcer and 1.30 (95% CI: 1.23–1.37) for patients with a duodenal ulcer (Fig. 1). Patients with gastric ulcer as a secondary registry diagnosis had a higher SIR than patients with gastric ulcer as the primary diagnosis compared to the general population, whereas it was less clear if there was a difference for duodenal ulcer patients (Table 2). Among gastric ulcer patients, men had a higher SIR of cancer than women. SIRs of any GI cancer in this ulcer cohort versus the general population was higher in patients younger than 40 years than in patients aged 65 years or older. For duodenal ulcer patients, there was no gender difference in GI cancer risk, and only patients diagnosed with an ulcer at ages 40–<65 years had an excess risk of any GI cancer (Table 2). SIRs were similar regardless of whether or patients received a gastroscopic examination during the hospital contact for an ulcer.

**Table 2:** Standardized incidence ratios (SIRs) (95% CIs) of cancer in patients with peptic ulcer disease, stratified by patient characteristics

	Gastric ulcer		Duodenal ulcer	
	O/E	SIR	O/E	SIR
All patients	1712/1243	1.38 (1.31–1.44)	1210/931	1.30 (1.23–1.37)
Women	780/600	1.30 (1.21–1.39)	440/350	1.26 (1.14–1.38)
Men	932/643	1.45 (1.35–1.55)	770/581	1.32 (1.23–1.42)
Age groups, years				
0–<40	29/13	2.15 (1.44–3.09)	15/16	0.93 (0.52–1.53)
40–<65	659/431	1.53 (1.42–1.65)	578/370	1.56 (1.44–1.69)
65+	1024/799	1.28 (1.20–1.36)	617/545	1.13 (1.05–1.23)
Calendar period, years				
1994–1998	713/537	1.33 (1.23–1.43)	545/445	1.23 (1.12–1.33)
1999–2003	544/389	1.40 (1.28–1.52)	369/291	1.27 (1.14–1.41)
2004–2008	359/254	1.41 (1.27–1.57)	222/159	1.40 (1.22–1.60)
2009–2013	96/64	1.51 (1.22–1.84)	74/37	1.98 (1.56–2.49)
Type of ulcer diagnosis				
Primary	1359/1013	1.34 (1.27–1.42)	1026/798	1.28 (1.21–1.37)
Secondary	353/231	1.53 (1.37–1.70)	184/133	1.39 (1.19–1.60)
Type of admission				
Inpatient	1227/865	1.42 (1.34–1.50)	909/682	1.33 (1.25–1.42)
Emergency room	98/63	1.56 (1.27–1.90)	29/22	1.32 (0.88–1.90)
Outpatient	387/315	1.23 (1.11–1.36)	272/227	1.20 (1.06–1.35)
Chronic obstructive				
Pulmonary disease				
Yes	129/81	1.59 (1.33–1.89)	87/58	1.51 (1.21–1.86)
No	1583/1162	1.36 (1.30–1.43)	1123/874	1.29 (1.21–1.36)
Chronic alcoholism				
Yes	216/67	3.25 (2.83–3.71)	174/50	3.47 (2.98–4.03)
No	1496/1177	1.27 (1.21–1.34)	1036/881	1.18 (1.11–1.25)
Severe liver disease				
Yes	51/11	4.70 (3.50–6.18)	34/6	5.49 (3.80–7.67)
No	1661/1233	1.35 (1.28–1.41)	1176/925	1.27 (1.20–1.35)
Diabetes				
Yes	145/92	1.58 (1.34–1.86)	87/16	1.43 (1.15–1.77)
No	1567/1152	1.36 (1.29–1.43)	1123/871	1.29 (1.22–1.37)
Obesity				
Yes	56/42	1.33 (1.01–1.73)	35/23	1.55 (1.08–2.15)
No	1556/1201	1.38 (1.31–1.45)	1175/909	1.29 (1.22–1.37)
Cardiovascular disease				
Yes	554/404	1.37 (1.26–1.49)	298/252	1.18 (1.05–1.33)
No	1158/839	1.38 (1.30–1.46)	912/679	1.34 (1.26–1.43)
Gastroscopy during same admission				
Yes	1266/934	1.35 (1.28–1.43)	931/710	1.31 (1.23–1.40)
No	446/309	1.44 (1.31–1.58)	279/222	1.26 (1.12–1.42)
Lower endoscopy during same admission				
Yes	109/86	1.26 (1.04–1.52)	80/57	1.41 (1.12–1.76)
No	1603/1157	1.39 (1.32–1.46)	1130/875	1.29 (1.22–1.37)

### Gastric cancer risk

Gastric ulcer patients had a higher occurrence of gastric cancer than duodenal ulcer patients, though absolute 1–5‐year risks were 0.25% versus 0.19% and the SIRs compared to the general population were 1.92 (95% CI: 1.65–2.23) versus 1.38 (95% CI: 1.12–1.68) (Fig. 1), respectively. Both gastric and duodenal ulcers were more strongly associated with non‐cardia gastric cancer than with cardia cancer (Fig. 1).

### Risk of other gastrointestinal cancers

The absolute 1–5‐year risks of other GI cancers among patients with gastric and duodenal ulcers were 1.9% and 1.8%, with similar risks in the two cohorts for the individual types of cancer. The highest absolute risk was observed for colon cancer, followed by oral and pharyngeal, gastric, pancreatic, liver, and rectal cancer, whereas risks of esophageal, small intestinal, anal, and gallbladder or biliary tract cancer were all <0.1%.

Although the overall SIR for other GI cancers was similarly increased for both gastric and duodenal ulcers (compared to the general population), there were some important differences by type of cancer. In general, the SIRs for smoking‐ and alcohol‐related cancers (e.g., oral and pharyngeal, pancreatic, liver, and colon cancer) were higher for patients with gastric ulcers than duodenal ulcers. The SIR of esophageal squamous cell carcinoma was similarly increased and more prominent than for adenocarcinoma, compared to the general population. The SIR of small intestinal cancer was higher among duodenal ulcer patients than gastric ulcer patients, whereas the SIR of gallbladder and biliary tract cancer was similarly increased in both cohorts. By contrast, one or more years after PUD diagnosis the SIRs for rectal cancer were decreased for both ulcer types. While patients with gastric ulcer had an increased risk of anal cancer, there was no association between duodenal ulcer and anal cancer (Fig. 1).

### Subgroup analyses

Patients with chronic alcoholism or severe liver disease and gastric or duodenal ulcers were at greater increased risk of GI cancers than the general population. Ulcer patients with chronic obstructive pulmonary disease or diabetes also had slightly higher excess relative risks than patients without these diseases, compared with the general population (Table 2). PUD patients who redeemed a prescription for NSAIDs, aspirin, or antacids in the year preceding the ulcer diagnosis had a lower cancer risk than patients who did not use these drugs, compared to the risk in the general population (Table 3). In contrast, PUD patients who had been treated with eradication therapy seemed to have a higher cancer risk than patients who did not receive eradication therapy prior to their ulcer diagnosis (Table 3).

**Table 3:** Standardized incidence ratios (SIRs) (with 95% CIs) of cancer in patients with peptic ulcer disease diagnosed between 2005 and 2013, stratified by medication use in the year preceding the ulcer diagnosis

	Gastric ulcer		Duodenal ulcer	
	O/E	SIR	O/E	SIR
NSAIDs				
Yes	222/174	1.27 (1.11–1.45)	153/104	1.48 (1.25–1.73)
No	133/75	1.76 (1.48–2.09)	85/49	1.74 (1.39–2.16)
PPI or H2–blockers				
Yes	123/104	1.19 (0.99–1.42)	82/57	1.44 (1.15–1.79)
No	232/146	1.59 (1.39–1.81)	156/96	1.63 (1.39–1.91)
Eradication therapy				
Yes	41/29	1.40 (1.00–1.90)	42/23	1.86 (1.34–2.52)
No	314/220	1.42 (1.27–1.59)	196/130	1.51 (1.31–1.74)

Aspirin and other nonsteroidal anti‐inflammatory drugs.

### Direct comparison of cancer risk in patients with gastric and duodenal ulcer

**Figure 2:**
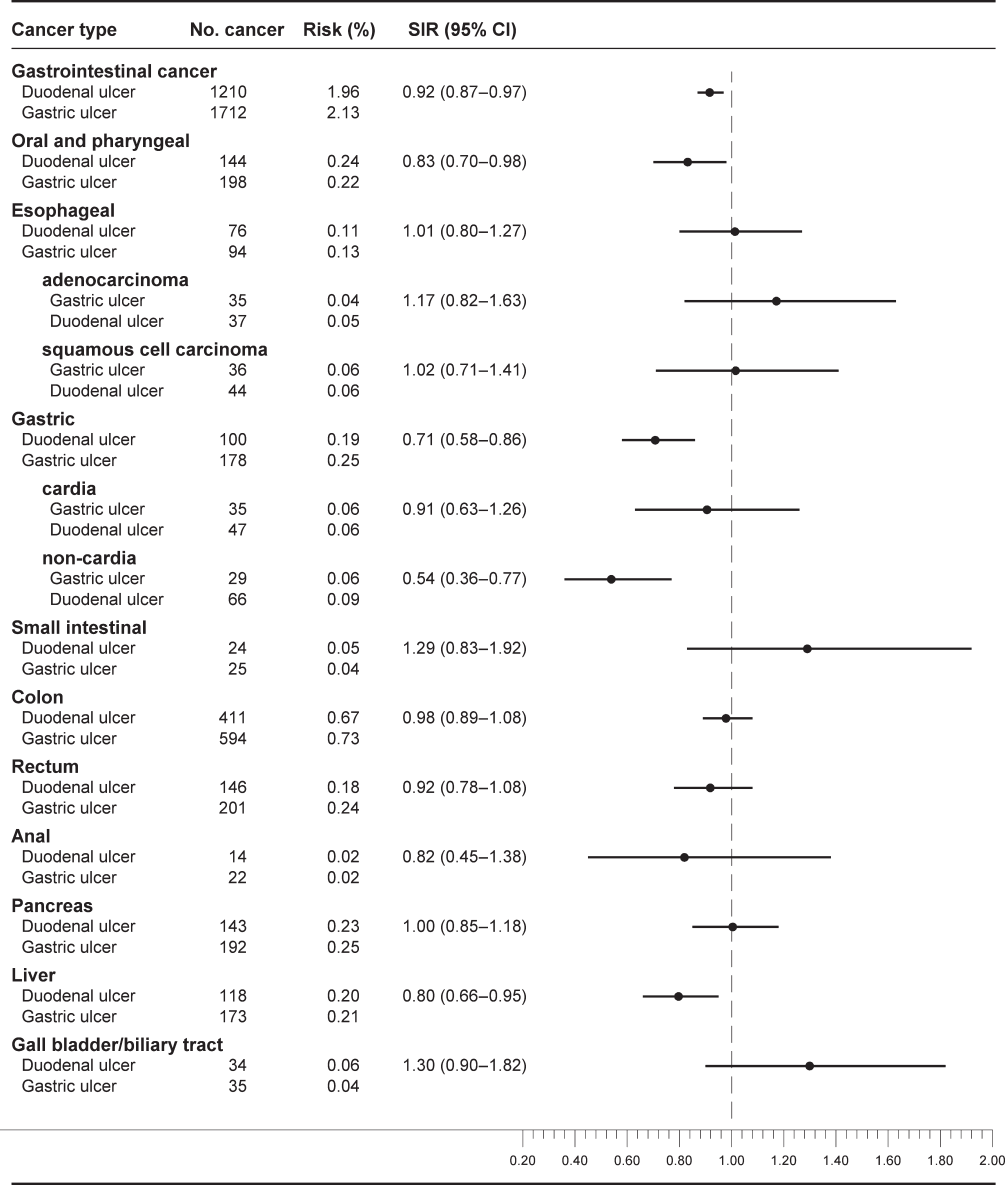
Standardized incidence ratios (SIRs) of cancer among duodenal ulcer patients compared to gastric ulcer patients.

When we directly compared the observed cancer risk among duodenal ulcer patients to the observed cancer risk in gastric ulcer patients as the reference group (Fig. 2), we found an 8% lower relative risk of GI cancer among the duodenal ulcer patients. The lower risk among duodenal compared to gastric ulcer patients stemmed primarily from an attenuated risk of oral and pharyngeal, gastric, liver, and rectal cancers. In contrast, duodenal ulcer patients had a higher risk of small intestinal cancer, and possibly a slightly higher risk of esophageal adenocarcinoma and gallbladder or biliary tract cancer compared with gastric ulcer patients (Fig. 2).

## Discussion

In this nationwide cohort study of 93,141 persons with a hospital‐based gastric or duodenal ulcer diagnosis, we observed an increased risk of gastric cancer as well as other GI cancers one or more years after the ulcer diagnosis, compared with the general Danish population. Site‐specific absolute cancer risks were similarly low in both PUD cohorts, and the direction of the associations with both gastric and other GI cancers was consistent among patients with the two ulcer types. As we found that patients with duodenal ulcers (closely H. pylori‐related) had a lower risk of several cancers than gastric ulcer patients, we question if H. pylori is an important player in the development of nongastric GI cancer.

H. pylori and its association with PUD and gastric cancer was discovered in the early 1980s 4. In more recent years, epidemiological studies have focused on potential associations between the bacteria and other GI cancers 7, 8, 9, but also on diseases related to systemic inflammation 26. The associations between H. pylori and other GI cancers remains controversial 6, but speculation includes both direct and indirect effects of the organism 6, 26. H. pylori infection may protect against esophageal adenocarcinoma through reduction in gastric acidity limiting precursor lesions in the lower esophagus 27, 28, but not squamous cell carcinoma 29, 30. The association with gastric adenocarcinoma appears to be restricted to increased risk of noncardia cancer 31. In contrast, H. pylori infection is associated with an increased risk of colorectal cancer 9, 32, 33, 34. Finally, evidence to support an association with other nongastric GI cancers is sparse, and null associations have been reported 8, 35, 36, 37, 38, 39, 40. In addition to H. pylori, several other environmental factors are associated with the risk of both PUD and cancer. Aspirin and other NSAIDs increase the risk of PUD but reduce the risk of gastric and colorectal cancer 11, 12, 13 (though such medications are likely discontinued at ulcer diagnosis). Use of proton‐pump inhibitors have been identified as predictors of increased gastric cancer risk, potentially reflecting a causal association or confounding by indication 41. Tobacco smoking is a common risk factor for both PUD 10, 16 and several GI cancers (oral, esophageal, gastric, pancreatic, and liver cancer) 14. Cirrhosis, a strongly alcohol‐related disease, also increases both risk of PUD 42 and GI cancer 14.

Based on the literature, we assumed that a higher proportion of duodenal than gastric ulcers would be H. pylori‐related 2, 3. Considering the proposed causal relation between H. pylori and cancer, we also expected to find a higher risk of various GI cancers (except for a lower risk of esophageal adenocarcinoma and gastric cardia cancer) among duodenal ulcer patients than among gastric ulcer patients. Instead, we found a lower risk of most cancers in patients with duodenal ulcers compared with gastric ulcers. The associations were not confined to patients with gastroscopically confirmed ulcer diagnoses. While the overall risk of esophageal cancer was increased in patients with duodenal ulcers, this stemmed mainly from an increased risk of squamous cell carcinoma, and we could not confirm a protective effect for adenocarcinoma in either of the cohorts. We confirmed that the increased risk of gastric cancer was mainly confined to noncardia cancer. However, whereas a previous study showed that duodenal ulcer was associated with decreased risk of gastric cancer 43, we found an increased risk for both gastric and duodenal ulcer patients. Our risk estimates for colon cancer were in agreement with studies reporting a modest increased risk of 20% 33. The decreased risk of rectal cancer may reflect diagnosis of prevalent cancers during lower endoscopy following bleeding among patients with ulcer disease (vs. no screening in the general population). Finally, we also noted that gastric ulcers conferred a higher risk than duodenal ulcers for GI cancers closely related to smoking and alcohol overuse.

Denmark is a welfare state with a tax‐supported and uniformly organized healthcare system. We used nationwide data, including both hospitalized patients and patients diagnosed in the outpatient setting, making selection bias unlikely. As well, the diagnoses in the registries used in our study have high validity 20, 22 and the positive predictive value for PUD has been reported to be 85% 1. We standardized on age, gender, and calendar year, but were unable to adjust for confounding factors. However, we found that the frequencies of covariates included in the analysis (coexisting morbidities, proxy measures of lifestyle factors, and NSAID use) were balanced for patients with gastric and duodenal ulcers. Thus, in our second analysis, in which we compared cancer risk among duodenal and gastric ulcer patients, the potential impact of confounding was reduced.

In conclusion, gastric and duodenal ulcers were associated with an increased risk of gastric and other GI cancers compared with the general Danish population. We speculate that H. pylori may play a less critical role in the development of nongastric GI cancer than previously hypothesized. Moreover, our findings may suggest that a follow‐up gastroscopy for oro‐pharyngeal, esophageal, and gastric cancer could be clinically relevant – at least among smokers. However, our data does not allow us to give recommendations on when such a repeat endoscopy should be performed; and finally to be cost‐beneficial such follow‐up procedure should also have a prognostic impact on the cancer survival.

## Conflict of Interest

The authors made no disclosures. The Department of Clinical Epidemiology, Aarhus University Hospital, receives funding for other studies from companies in the form of research grants to (and administered by) Aarhus University. None of those studies have any relation to this study.

## Appendix A1 Diagnosis codes (ICD‐8 and ICD‐10) used in the study

### Cohort with ulcer disease

Gastric ulcer: ICD‐10: K25.

Duodenal ulcer: ICD‐10: K26.

### Cancer

Gastrointestinal cancers, classified by ICD‐10: combined and separately: Oral and pharyngeal [tongue (C01‐02), oral cavity (C03‐06), salivary gland (C07‐08), pharyngeal tonsil/cavity (C09‐10), other parts of pharynx (C12‐13)], esophagus (C15): esophageal adenocarcinoma (C15 with morphology codes 81403, 81433, 81443, 82113, 82303, 82603, 84303, 84803, 84813, 84903) and squamous cell carcinoma (C15 with morphology codes 80703 or 80713), gastric (C16): cardia C16.0 and noncardia 16.1–16.6, small intestine (C17), colon including rectosigmoid (C18–19), rectum (C20), anal (C21), liver (C22), gallbladder and biliary tract (C23–24), and pancreas (C25).

### Covariates

#### Chronic obstructive pulmonary disease

ICD‐8: 491, 492, ICD‐10: J41–J44.

#### Chronic alcoholism

ICD‐8: 291.00–291.99, 303.00–303.99, 571.09, 571.10, 577.10, ICD–10: E24.4, E52.9A, F10.1, F10.2–10.9, G31.2, G62.1, G72.1, I42.6, K29.2, K.70, K85.2, K86.0, T50.0A, Z50.2, Z71.4, Z72.1.

#### Severe liver disease

ICD–8: 070.00, 070.02, 070.04, 070.06, 070.08, 573.00, 456.00–456.09, ICD–10: B15.0, B16.0, B16.2, B19.0, K70.4, K72, K76.6, I85.

#### Diabetes

ICD–8: 249.00‐249.09, 250.00‐250.09, ICD‐10: E10, E11, E14.

#### Obesity

ICD–8: 277.99, ICD–10: E65, E66.

#### Cardiovascular disease

ICD–8: 393–397, 400–404, 410–414, 424, 427.09–427.11, 427.19, ICD–10: I05–08, I10–15, I20–25, I34–37, I39, I50, I51.1A.

### Anatomical Therapeutic Chemical (ACT) codes used in the study

#### NSAIDs

Low‐ and high‐dose aspirin (B01AC06, N02BA01, N02BA51); Selective cyclooxygenase‐2 inhibitors: M01AH01, M01AH02, M01AH03, M01AH05, M01AC05, M01AB05, M01AC06; other NSAIDs: All other codes within group M01A. Proton pump inhibitors (PPI): A02BC and Histamin2 receptor antagonist (H2RA): A02BA. *H. pylori* eradication (amoxicillin, clarithromycin, and metronidazole): J01CA04, J01FA09, and P01AB01.

## References

[1] Lassen, A. , J. Hallas , and O. B. Schaffalitzky de Muckadell . 2006 Complicated and uncomplicated peptic ulcers in a danish county 1993–2002: a population‐based cohort study. Am. J. Gastroenterol. 101:945–953. 16573778 10.1111/j.1572-0241.2006.00518.x

[2] Gisbert, J. P. , and X. Calvet . 2009 Review article: helicobacter pylori‐negative duodenal ulcer disease. Aliment. Pharmacol. Ther. 30:791–815. 19706147 10.1111/j.1365-2036.2009.04105.x

[3] Musumba, C. , A. Jorgensen , L. Sutton , D. Van Eker , J. Moorcroft , M. Hopkins , et al. 2012 The relative contribution of NSAIDs and helicobacter pylori to the aetiology of endoscopically‐diagnosed peptic ulcer disease: observations from a tertiary referral hospital in the UK between 2005 and 2010. Aliment. Pharmacol. Ther. 36:48–56. 22554233 10.1111/j.1365-2036.2012.05118.x

[4] Marshall, B. J. , and J. R. Warren . 1984 Unidentified curved bacilli in the stomach of patients with gastritis and peptic ulceration. Lancet 1:1311–1315. 6145023 10.1016/s0140-6736(84)91816-6

[5] Palli, D. , G. Masala , G. Del Giudice , M. Plebani , D. Basso , D. Berti , et al. 2007 CagA+ helicobacter pylori infection and gastric cancer risk in the EPIC‐EURGAST study. Int. J. Cancer 120:859–867. 17131317 10.1002/ijc.22435

[6] Tatishchev, S. F. , C. Vanbeek , and H. L. Wang . 2012 Helicobacter pylori infection and colorectal carcinoma: is there a causal association? J. Gastrointest. Oncol. 3:380–385. 23205318 10.3978/j.issn.2078-6891.2012.058 PMC3492471

[7] Vineis, P. , P. Crosignani , C. Sacerdote , A. Fontana , G. Masala , L. Miligi , et al. 1999 Hematopoietic cancer and peptic ulcer: a multicenter case‐control study. Carcinogenesis 20:1459–1463. 10426792 10.1093/carcin/20.8.1459

[8] Risch, H. A. , H. Yu , L. Lu , and M. S. Kidd . 2010 ABO blood group, helicobacter pylori seropositivity, and risk of pancreatic cancer: a case‐control study. J. Natl Cancer Inst. 102:502–505. 20181960 10.1093/jnci/djq007 PMC2902822

[9] Wu, Q. , Z. P. Yang , P. Xu , L. C. Gao , and D. M. Fan . 2013 Association between helicobacter pylori infection and the risk of colorectal neoplasia: a systematic review and meta‐analysis. Colorectal Dis. 15:e352–e364. 23672575 10.1111/codi.12284

[10] Kurata, J. H. , and A. N. Nogawa . 1997 Meta‐analysis of risk factors for peptic ulcer. nonsteroidal antiinflammatory drugs, helicobacter pylori, and smoking. J. Clin. Gastroenterol. 24:2–17. 9013343 10.1097/00004836-199701000-00002

[11] Wu, C. Y. , M. S. Wu , K. N. Kuo , C. B. Wang , Y. J. Chen , and J. T. Lin . 2010 Effective reduction of gastric cancer risk with regular use of nonsteroidal anti‐inflammatory drugs in helicobacter pylori‐infected patients. J. Clin. Oncol. 28:2952–2957. 20479409 10.1200/JCO.2009.26.0695

[12] Akre, K. , A. M. Ekstrom , L. B. Signorello , L. E. Hansson , and O. Nyren . 2001 Aspirin and risk for gastric cancer: a population‐based case‐control study in sweden. Br. J. Cancer 84:965–968. 11286478 10.1054/bjoc.2001.1702 PMC2363844

[13] Friis, S. , A. H. Poulsen , H. T. Sorensen , A. Tjønneland , K. Overvad , U. Vogel , et al. 2009 Aspirin and other non‐steroidal anti‐inflammatory drugs and risk of colorectal cancer: a danish cohort study. Cancer Causes Control 20:731–740. 19122977 10.1007/s10552-008-9286-7

[14] Secretan, B. , K. Straif , R. Baan , Y. Grosse , F. El Ghissassi , V. Bouvard , et al. 2009 A review of human carcinogens–part E: tobacco, areca nut, alcohol, coal smoke, and salted fish. Lancet Oncol. 10:1033–1034. 19891056 10.1016/s1470-2045(09)70326-2

[15] Sonnenberg, A. , and I. H. Wasserman . 1995 Associations of peptic ulcer and gastric cancer with other diseases in US veterans. Am. J. Public Health 85:1252–1255. 7661233 10.2105/ajph.85.9.1252 PMC1615583

[16] Kato, I. , A. M. Nomura , G. N. Stemmermann , and P. H. Chyou . 1992 A prospective study of gastric and duodenal ulcer and its relation to smoking, alcohol, and diet. Am. J. Epidemiol. 135:521–530. 1570818 10.1093/oxfordjournals.aje.a116319

[17] Aro, P. , T. Storskrubb , J. Ronkainen , E. Bolling‐Sternevald , L. Engstrand , M. Vieth , et al. 2006 Peptic ulcer disease in a general adult population: the kalixanda study: a random population‐based study. Am. J. Epidemiol. 163:1025–1034. 16554343 10.1093/aje/kwj129

[18] Ministry of health and prevention . 2012 eHealth in Denmark. Available at: http://Www.sum.dk/~/media/Filer%20-%20Publikationer_i_pdf/2012/sundheds-IT/Sundheds_IT_juni_web.ashx (accessed June 25, 2015).

[19] Pedersen, C. B. 2011 The danish civil registration system. Scand. J. Public Health 39(7 Suppl.):22–25. 21775345 10.1177/1403494810387965

[20] Schmidt, M. , S. A. Schmidt , J. L. Sandegaard , V. Ehrenstein , L. Pedersen , and H. T. Sorensen . 2015 The danish national patient registry: a review of content, data quality, and research potential. Clin. Epidemiol. 7:449–490. 26604824 10.2147/CLEP.S91125 PMC4655913

[21] Johannesdottir, S. A. , E. Horvath‐Puho , V. Ehrenstein , M. Schmidt , L. Pedersen , and H. T. Sorensen . 2012 Existing data sources for clinical epidemiology: the danish national database of reimbursed prescriptions. Clin. Epidemiol. 4:303–313. 23204870 10.2147/CLEP.S37587 PMC3508607

[22] Storm, H. H. , E. V. Michelsen , I. H. Clemmensen , and J. Pihl . 1997 The danish cancer registry–history, content, quality and use. Dan. Med. Bull. 44:535–539. 9408738

[23] Kim, H. T. 2007 Cumulative incidence in competing risks data and competing risks regression analysis. Clin. Cancer Res. 13(2 Pt 1):559–565. 17255278 10.1158/1078-0432.CCR-06-1210

[24] Rothman, K. J. , S. Greenland , and T. L. Lash . 2008 Modern epidemiology. 3rd ed Lippincott Williams & Wilkins, Philedelphia.

[25] Breslow, N. E. , and N. E. Day . 1987 Statistical methods in cancer research. Volume II–the design and analysis of cohort studies. IARC Sci. Publ. 82:1–406. 3329634

[26] Franceschi, F. , G. Zuccala , D. Roccarina , and A. Gasbarrini . 2014 Clinical effects of helicobacter pylori outside the stomach. Nat. Rev. Gastroenterol. Hepatol. 11:234–242. 24345888 10.1038/nrgastro.2013.243

[27] Richter, J. E. , G. W. Falk , and M. F. Vaezi . 1998 Helicobacter pylori and gastroesophageal reflux disease: the bug may not be all bad. Am. J. Gastroenterol. 93:1800–1802. 9772034 10.1111/j.1572-0241.1998.00523.x

[28] Fischbach, L. A. , D. Y. Graham , J. R. Kramer , M. Rugge , G. Verstovsek , P. Parente , et al. 2014 Association between helicobacter pylori and barrett's esophagus: a case‐control study. Am. J. Gastroenterol. 109:357–368. 24419485 10.1038/ajg.2013.443 PMC4046944

[29] Rokkas, T. , D. Pistiolas , P. Sechopoulos , I. Robotis , and G. Margantinis . 2007 Relationship between helicobacter pylori infection and esophageal neoplasia: a meta‐analysis. Clin. Gastroenterol. Hepatol. 5:1413–1417, 1417.e1‐2. 17997357 10.1016/j.cgh.2007.08.010

[30] Islami, F. , and F. Kamangar . 2008 Helicobacter pylori and esophageal cancer risk: a meta‐analysis. Cancer Prev. Res. (Phila.) 1:329–338. 19138977 10.1158/1940-6207.CAPR-08-0109 PMC3501739

[31] Helicobacter and Cancer Collaborative Group . 2001 Gastric cancer and helicobacter pylori: a combined analysis of 12 case control studies nested within prospective cohorts. Gut 49:347–353. 11511555 10.1136/gut.49.3.347 PMC1728434

[32] Zhao, Y. S. , F. Wang , D. Chang , B. Han , and D. Y. You . 2008 Meta‐analysis of different test indicators: helicobacter pylori infection and the risk of colorectal cancer. Int. J. Colorectal Dis. 23:875–882. 18506454 10.1007/s00384-008-0479-z

[33] Wang, F. , M. Y. Sun , S. L. Shi , and Z. S. Lv . 2013 Helicobacter pylori infection and normal colorectal mucosa ‐ adenomatous polyp ‐ adenocarcinoma sequence: a meta‐analysis of 27 case‐control studies. Colorectal Dis. 16:246–252. 23692360 10.1111/codi.12290

[34] Zumkeller, N. , H. Brenner , M. Zwahlen , and D. Rothenbacher . 2006 Helicobacter pylori infection and colorectal cancer risk: a meta‐analysis. Helicobacter 11:75–80. 16579836 10.1111/j.1523-5378.2006.00381.x

[35] Mishra, R. R. , M. Tewari , and H. S. Shukla . 2011 Helicobacter pylori and pathogenesis of gallbladder cancer. J. Gastroenterol. Hepatol. 26:260–266. 21261714 10.1111/j.1440-1746.2010.06435.x

[36] Garcia, A. , Y. Feng , N. M. Parry , A. McCabe , M. W. Mobley , K. Lertpiriyapong , et al. 2013 Helicobacter pylori infection does not promote hepatocellular cancer in a transgenic mouse model of hepatitis C virus pathogenesis. Gut. Microbes 4:577–590. 23929035 10.4161/gmic.26042 PMC3928167

[37] Verhoef, C. , R. G. Pot , R. A. de Man , P. E. Zondervan , E. J. Kuipers , J. N. IJzermans , et al. 2003 Detection of identical helicobacter DNA in the stomach and in the non‐cirrhotic liver of patients with hepatocellular carcinoma. Eur. J. Gastroenterol. Hepatol. 15:1171–1174. 14560149 10.1097/00042737-200311000-00004

[38] Xuan, S. Y. , N. Li , X. Qiang , R. R. Zhou , Y. X. Shi , and W. J. Jiang . 2006 Helicobacter infection in hepatocellular carcinoma tissue. World J. Gastroenterol. 12:2335–2340. 16688821 10.3748/wjg.v12.i15.2335 PMC4088066

[39] Yu, G. , G. Murphy , A. Michel , S. J. Weinstein , S. Männistö , D. Albanes , et al. 2013 Seropositivity to helicobacter pylori and risk of pancreatic cancer. Cancer Epidemiol. Biomarkers Prev. 22:2416–2419. 24089457 10.1158/1055-9965.EPI-13-0680 PMC3858455

[40] Bosetti, C. , E. Lucenteforte , P. M. Bracci , E. Negri , R. E. Neale , H. A. Risch , et al. 2013 Ulcer, gastric surgery and pancreatic cancer risk: an analysis from the international pancreatic cancer case‐control consortium (PanC4). Ann. Oncol. 24:2903–2910. 23970016 10.1093/annonc/mdt336 PMC3811904

[41] Poulsen, A. H. , S. Christensen , J. K. McLaughlin , R. W. Thomsen , H. T. Sørensen , J. H. Olsen , et al. 2009 Proton pump inhibitors and risk of gastric cancer: A population‐based cohort study. Br. J. Cancer 100:1503–1507. 19352380 10.1038/sj.bjc.6605024 PMC2694435

[42] Luo, J. C. , H. B. Leu , M. C. Hou , C. C. Huang , H. C. Lin , F. Y. Lee , et al. 2012 Cirrhotic patients at increased risk of peptic ulcer bleeding: a nationwide population‐based cohort study. Aliment. Pharmacol. Ther. 36:542–550. 22817655 10.1111/j.1365-2036.2012.05225.x

[43] Hansson, L. E. , O. Nyren , A. W. Hsing , R. Bergström , S. Josefsson , W. H. Chow , et al. 1996 The risk of stomach cancer in patients with gastric or duodenal ulcer disease. N. Engl. J. Med. 335:242–249. 8657240 10.1056/NEJM199607253350404

